# Enhancing productivity through work study - A case of electric power pole cross arm fabrication

**DOI:** 10.1016/j.heliyon.2024.e32868

**Published:** 2024-06-12

**Authors:** Yonas Mitiku Degu

**Affiliations:** Mechanical Engineering Program, Faculty of Mechanical and Industrial Engineering, Institute of Technology, Bahir Dar University, Ethiopia

**Keywords:** Work study, Productivity, Production time, Electric pole cross arm, Work measurement

## Abstract

In the area of globalization any manufacturing industry must be competent in terms of productivity, quality, cost and delivery. A fundamental improvement in production is necessary to succeed in the international markets. Work study is one of the earliest scientific management methods used to determine the best way to perform production tasks in order to reduce idle time and worker fatigue as a result, increase productivity. Kombolcha Steel Products Industry, the member of MIDROC Ethiopia Technology Group, producing a variety of metal and engineering products, such as steel poles, steel structures, structural hot dip galvanization, corrugated sheet metal, etc. The company's main focus was meeting the delivery deadlines, with little attention to implementing standard working method and procedures during the production. The inefficient method of piercing the holes in the u-channels to fabricate the electric pole cross arms is causing a drop in productivity.

The primary objective of the current study is to identify the bottlneck in the process of electric power poles cross arms fabrication, to set standard time for the process and reduce the cycle time. Time and method studies were used to identify the flaws in the current fabrication process and layout design. The efficient technique to manufacture the product has been replaced by redesigning and implementing a new layout. This arrangement accelerated the process by reducing the idle time for the three punching machines that undertake the piercing operations. The modified layout raised production from the target daily production of 420 pieces to 720 pieces in 8 h, resulting in a 71 % reduction in the previous work cycle from 65 to 39 s. After a time and method study, a simple change in the layout with no capital investment not only increased the company's productivity and profitability, but it also reduced worker fatigue due to extra material handling operations of raw materials and intermediate products.

## Highlights

• Work study is found to be effective method to enhancing industry productivity.
• No capital was invested to implement the new layout design.
• The new configuration enhanced company's productivity by 71 %.
• Reduction of work cycle from 65 to 39 s is achieved by the new setup.
• Fatigue among the operators working on the first and the last machines have significantly decreased.

## 1 Introduction

The current state of the global industry is challenging due to the quick changes in the global competitive landscape, market dynamics, and technological advancements. As a result, they are putting in a lot of effort to meet customer demand and maintain their competence in their particular product area. This is valid for Ethiopia's manufacturing sectors, which strive for productivity, speed, and quality competitiveness [1,2]. The productivity enhancement in a manufacturing industry can be achieved in several ways, including capital investments, technological advancements, and efficient use of human resources. Enhancing productivity through effective use of human resources primarily addresses the need to meet specific needs that spur individuals to work diligently. On the other hand, productivity improvement through effective use of resources mainly focuses on the need for waste minimization at all stages of production. Productivity by undertaking technological innovation and development is primarily concerned with work study techniques. Various scholars have defined work study as an entire set of techniques for simplifying, standardizing, and measuring of work. Work study may require little or no capital investment. Productivity enhancement through capital investment usually corresponds to a technological shift. Transformation in technology comes into play when it is failed to meet customer requirements in terms of both quantity and quality, and when the technology is not any longer economically viable. However, this change requires a significant capital investment as well as time [3].

Work study is an organized assessment of the methods of carrying out activities in order to raise resource efficiency and set performance standards for the activities being performed. It constitutes of method study and work measurement [4,5].

Due to the nature of very little or no capital investment work study methods is preferred at the first stage to enhance the productivity and standardizing of work in industries. The case company Kombolcha Steel Products Industry (KOSPI) - Gelan is a part of the MIDROC Ethiopia Technology Group, which is situated on the outskirts of Ethiopia's capital city, Addis Abeba was founded in 1999.

KOSPI - Gelan is a company that produces and distributes a range of engineering and metal products used in the different sectors. These include cargo bodies, storage tanks, pre-engineered steel buildings, wire products, corrugated and ribbed sheets, structural hot dip galvanization, electric pole cross arms, and other engineered metal products.

Over the course of the one-month author externship, the industry was assessed to identify obstacles to the company's competitiveness and productivity. It was discovered that several technical issues were identified in different departments of the company.

Among them, the electric pole crosses arm production processes were chosen as a showcase due to inconsistent production performance, which necessitates work standardization that could boost productivity of the company.

**Fig. 1:**
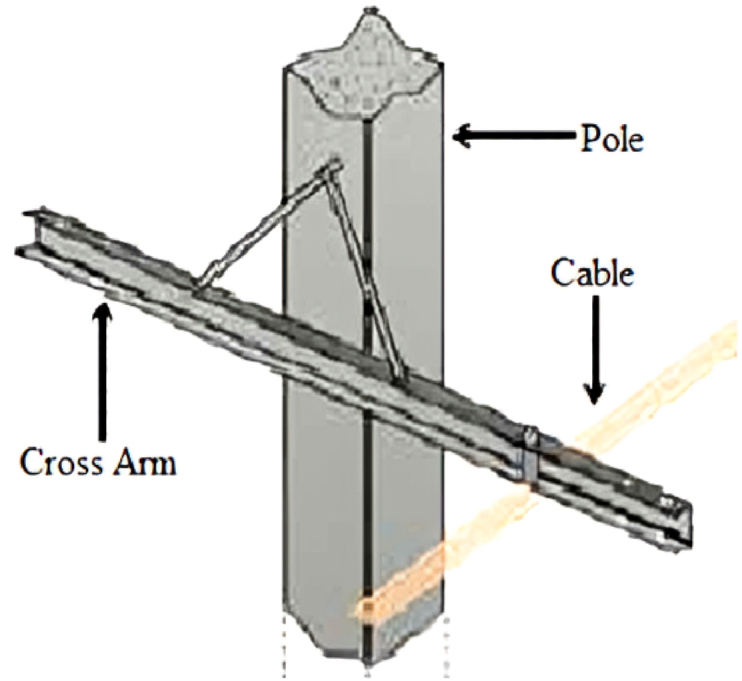
Cross arm attached to the electrical power pole [6].

**Fig. 2:**
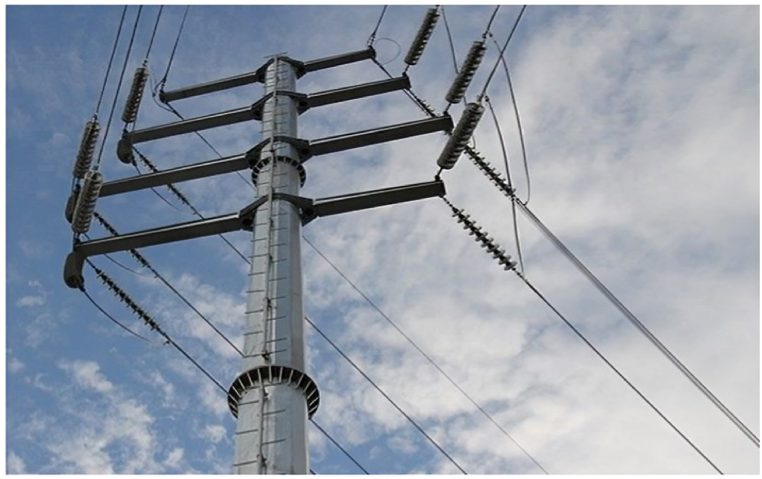
Single power pole with multiple cross arms [7].

Most electrical power structures are made up of two major components: cross arms and poles. The cross arm (Fig. 1.) is the bar connected to the main pole that carry either electrical power wires or communication cables. A single power pole may have multiple cross arms attached to it, as illustrated in Fig. 2.

## 2 Literature review

**Fig. 3:**
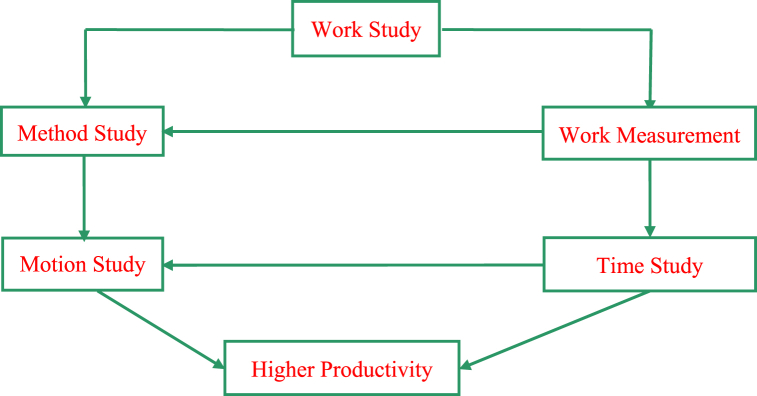
Conceptual model for work study and productivity enhancement [1,10,15].

Work study is the most popular tool for identifying processes that take extra time and thus reduce productivity. It involves systematically recording the time spent in various operations, critical analysis, and the development of the most efficient process to minimize idle time, thereby increasing productivity and decreasing worker fatigue [[8], [9], [10], [11]]. Work study methods are widely used in a variety of industries for improving productivity and growth. It is divided into two sections: method study and work measurement. Further more work measurement can be achieved via time study and motion study [12,13]. Thus, work study is particularly concerned with productivity [13]. Method studies are commonly used to improve a work method, which is especially important for new jobs [8]. When applied to existing jobs, its focus will be on finding efficient methods to do the job that are economical, safe, demand less human effort, and take less make-ready time. Superior efficiency is attained through improved layout and workplace design, improved and efficient work procedures, effective utilization of human resources, machinery, materials, and generally improved final product design or specification [10,12,14]. In order to improve a situation, both method study and work measurement are used to assess human effort in all of its contexts [11,13,15] and lead systematically to the investigation of all the factors that affect the economy and efficiency of the situation under review. Fig. 3 depicts the conceptual model of the relationship between work study and productivity. Time study, on the other hand, provides the standard time, which is the time needed by worker to finish a job by the standard method [16]. When market interest is high and manufacturers require more yield in a short period of time, ergonomics discovered an extraordinary need [17].

By implementing work study, productivity can be increased to a respectable extent. Increasing productivity through capital expenditures essentially involves a change in technology [3]. We can achieve superior output at lower costs with greater quality and thus increase productivity by implementing method study and time study in any organization.

The objective of the present study is to identify the current challenge in the production line of the electric power pole cross arm and to develop a new system with the aid of the work study technique in order to enhance productivity by eliminating non-value added time for the process.

## 3 Materials and methods

### 3.1 Materials

**Fig. 4:**
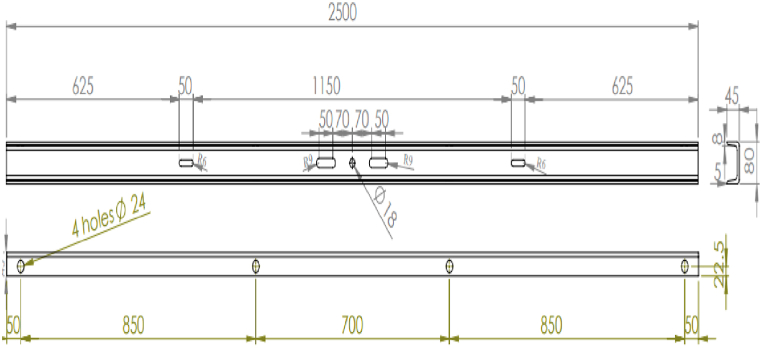
Geometrical dimension of 33 KV electric power pole cross arm.

U-channel structural carbon steels are used for the fabrication of electric power pole cross arm, which has dimensional requirements set by the customer so as to withstand the load imposed by 33 KV electric lines and the working environment. The detail geometrical dimensions are given in Fig. 4, which mainly contains slots and holes. The slots and holes are designed to attaching insulators to be suspended as well as to attach with pole.

### 3.2 Machines and instruments

**Fig. 5:**
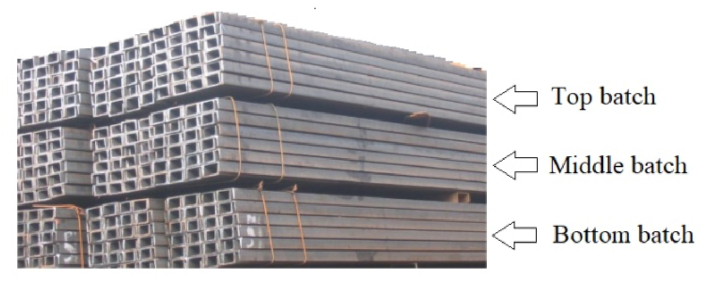
Bach of 2.5 m long U channels ready for fabrication electric power pole cross arm.

In order to achieve the desired feature as shown in Fig. 4, three mechanical punching machines with capacities of 35 tons, 50 tons, and 75 tons each equipped with a variety of cutting tools were used to form holes and slots on the u-channels (Fig. 5). In addition, measuring tools tape meter and stopwatch were used for measuring the layout and time, respectively.

### 3.3 Methodology

A conceptual framework is created in order to carry out the research work in an organized manner. A descriptive research design was employed while quantitative and qualitative approaches were used. Data were gathered through work sampling, direct observations, and interviews.

i) Structured interviews

Discussion with the management team and employees is required to get their thoughts on the issue at hand and to foster cooperation for the implementation of solutions.

The sample questions used for interviewing the production manager, production supervisors, and general workers are listed in Table 1.

ii) Observation

**Table 1:** Leading questions for interviews.

S·N.	Leading questions posed to employees at various levels
**1.**	What are the steps involved in the fabrication of an electric pole cross arm?
**2.**	Are all of the steps necessary to complete the task?
**3.**	How long does it typically take to complete each step?
**4.**	Is there flexibility in the order of tasks?
**5.**	Is it possible to combine a particular step in the process with other steps?
**6.**	Is there an alternative methodology of handling each task?
**7.**	Does the worker need a higher level of skill?
**8.**	What kinds of skill development programs does the company run?
**9.**	How does machine downtime affect productivity?
**10.**	Which maintenance techniques are being applied?

The company was first visited in order to determine which products or processes had the greatest potential for improvement at the lowest possible cost of implementation. The researcher observed the electric power pole cross arm fabrication process as well as the workers' actions firsthand. This method was preferred because it allowed the researchers to observe and record events without relying on the willingness or ability of the respondents to report. Because the procedures and techniques were thoroughly examined, human error will be reduced. In this regard, problems will be identified, and new methods for the selected activity will be developed.

iii) Work sampling

Work sampling is a research technique where workers are observed at random times and documenting their activities. It was observed that the electric power pole cross arm fabrication process was taking more time than expected based on the company daily report sheets and on-site observations.

A time study must be carried out in order to compare new accomplishments to the outcomes obtained prior to the operation's modification and serve as a benchmark. Furthermore, a discussion will be held with the staff and the company management body regarding the success of the implementation and what needs to be improved further for the future.

The sample size (n) for the observations to be conducted was determined using the confidence level determined on the margin of error that could allow for these observations to be done. Most commonly 95 % confidence interval is used in such researches [18]. A specific observation was deemed accurate within ±5 %, or 10 %, 95 % of the time.

The prior research study, which computes the ratio of survey respondents who selected the pertinent response, is what determines the sample proportion (p).

Since we don't have any data from earlier research or pilot studies, the typical proportion value in this research sample is 50 %. Equation (1) is used to determine the required sample size for the investigation.

(1) $$n=\frac{\lambda^{2}p\;\left(1-p\right)}{\;\varepsilon^{2}}$$

where, n = Sample size.

λ = Value based on confidence level (Table 2).

**Table 2:** Lambda (λ) values for different confidence level [18,19].

Confidence Level	Lambda (λ) values
90 %	1.65
95 %	1.96
99 %	2.58

p = Sample proportion

ε = Margin of error.

For this study the sample size is:

$$n=\frac{{\left(1.96\right)}^{2}*0.5*\left(1-0.5\right)}{\;{\left(0.1\right)}^{2}}=96\approx 100$$

iv) Method study

Method study is the methodical recording and thorough evaluation of existing and proposed work methods in order to develop and apply easier and more effective methods while simultaneously lowering costs. The flow process chart and flow diagrams are very simple and effective tools of method study.

The following steps are followed in the method study:

1. Select the work to be analyzed
2. Record every detail regarding the current method using charts, diagrams, layout models etc
3. Examine the recorded facts critically and objectively considering the purpose, place, sequence, person and means
4. Develop the most cost-effective method for satisfying plant requirements, taking into account technical viability, economics, human factors, and practicability.
5. Install the new method as standard practice
6. Maintain the newly developed method by systematic and periodic checks to find any deviations observed, modify or eliminate deviations or improvements through changes

v) Time study

Time Study is the analysis of a specific job by a qualified worker to determine the most efficient method in terms of time and effort. In the manufacturing industry, time study is used to standardize tasks, set productivity targets for skilled workers, eliminate waste, improve process consistency, reduce variability, and enhance quality [20]. The time study also assists in determining manpower requirements. The time was recorded with the help of stopwatch since it is easier and faster in data recording.

The procedure for carrying out a time study is as follows [21]:

1. Identify the task and the operation to be timed
2. Determine the number of cycles to study
3. Choose eligible workers
4. Explain the time study details to the workers and supervisor
5. Conduct observations and record time using stopwatch time study
6. Repeat the above steps for number of observations as determined in earlier steps.
7. Analyze workers’ performance
8. Explain to the worker the improved working procedure and use of tools to do the job
9. Repeat the above steps for number of observations as determined in earlier steps
10. Use the data to calculate standard time

Descriptive statistics are used to get a sense of the distribution of time study data. Thus, in this study the mean and standard deviation parameters are computed.

A total of one hundred samples before and after the new setup to investigate the effect of the later setup on productivity.

The first ten samples of time study were averaged and the mean values were taken as a single experimental test, thus, it will reduce the variability of the test data [22].

## 4 Results and discussion

### 4.1 Process study

Identify inefficient fabrication processes that could be resolved at a low cost and with a substantial effect on the company's productivity was the concern of the study. In addition the machine layout, the number of material handling operations, and the amount of human fatigue associated with all of the operations was assessed. A preliminary survey was conducted on different department of the industry's fabrication procedures for various engineering products. The manufacturing process for electric power pole cross arms were discovered to have the greatest potential for productivity enhancement and work study implementation.

To make the cross arms, the company uses two different lengths of u-channel: the first type, which is supplied by a nearby steel manufacturing industry and has a required final dimension of 2.5 m. The second kind, however, is 6 m long unlike to the first type which requires cutting to the size before starting the punching operation.

In order to start the manufacturing process, the U-channels are transported by forklift from the raw material store to the fabrication shop. It has been identified that the workers are unaware of the proper placement of the raw materials in the fabrication shop. Furthermore, as shown in Fig. 5, up to three batches (each 100 pieces) of raw material are stacked, causing the height to rise to a height that makes it difficult for the operators to unload and feed to the punching machines. Though the company uses four punching machines to form the final product, the investigation is limited to the three machines because the fourth machine is located considerably farther away and the process is not carried out sequentially. It also necessitates a major revision of the layout design, which demands a significant amount of capital that the company may be unprepared to bear.

**Fig. 6:**
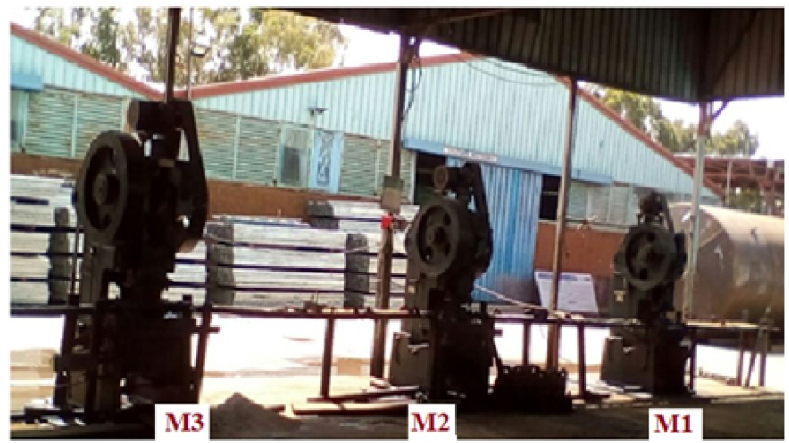
The existing punching machines and jigs layout [Source:- Photo taken by Menberu Zeleke, FMIE, BIT-BDU].

The primary tasks involved in producing the electric pole cross arm are listed in Table 3. The U-channels are fed into machine M1, which is followed by machines M2 and M3 which is used to perform a series of piercing operations as indicated in Fig. 6. Jigs and fixtures are used to locate the exact location of the operation to be executed by each machine.

**Table 3:** Core activities in the manufacturing processes of electric pole cross arm.

Activities	Description
Loading	Loading of the raw material
Piercing	Piercing of the center hole (φ18mm) by using M_1_
Piercing	Piercing of the smaller slots (R6) at two locations using M_2_
Piercing	Piercing of the larger slots (R9) at two locations using M_3_
Unloading and stacking	Unloading and stacking of the work piece

Following completion of the punching processes in all the three machines the intermediate product is stacked next to M3. It was discovered that the stacking of intermediate product materials suffers the same problem of placing randomly as raw materials. The product's quality was checked by the company quality department on a regular basis for every 100 pieces. The intermediate products were lifted by fork lift to the fourth punching machine which is situated in a different zone of the work shop (not covered under this study), where the remaining eight holes are pierced along the sides of the u-channels (Fig. 4). Upon the completion of all piercing, the electric pole cross arm was taken to the hot dip galvanizing section for zinc coating.

**Fig. 7:**
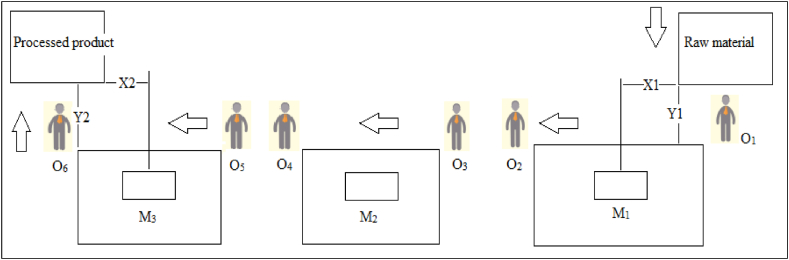
Processing sequence of operations and machines layout diagram.

The fabrication of the electric pole cross arm using the three machines requires six operators (O1, O2, …,O6). All the operators chosen are male and the age ranges from 21 to 25 years old. Each operator's duties and responsibilities are provided in Table 4. The work process flow diagram Fig. 7 allows for a thorough understanding of the fabrication process.

**Table 4:** Operators and their responsibility.

Individual	Duties and Responsibility
**Operator 1 (O1)**	Loading of raw material to M1 along with O2, pushing into M1, placing stopper on the jig to pierce the center hole
**Operator 2 (O2)**	Loading of raw material to M1 along with O1, pulling to M1, operating M1 with his foot and pushing the work piece to M2
**Operator 3 (O3)**	Assist pulling the work piece from M1, pushing the work piece into M3 and placing stopper on the jig for piercing the two R6 slots
**Operator 4 (O4)**	Pulling the work piece from M2, pushing the work piece to M3 and operating M2 with his foot.
**Operator 5 (O5)**	Assist pulling of work piece from M2, pushing into M3 and placing stopper on the jig for piercing the two R9 slots.
**Operator 6 (O6)**	Work piece pulling from M3, unloading and stacking intermediate product along with O5, and operating M3 with his foot.

The study identifies the manufacturing process bottlenecks, suggests a new layout for raw materials and intermediate products, and conducts time and method studies to improve industry productivity by minimizing worker fatigue and eliminating unnecessary material handling operations. It was discovered during the visit to the workshop and conversations with the manager that the inefficient time associated with non-value-adding movement is greater on operators (O1, O2, O5 and O6).

Productivity measurement frequently involves comparing inputs and outputs to generate a productivity index, which can be calculated qualitatively using equation (2), equation (3) and equation (4) [23].

(2) $$\text{Productivity}=\frac{\text{Output}}{\text{Input}}$$

(3) $$\text{Efficiency}=\frac{\text{Minutes}\;\text{output}}{\text{Minutes}\;\text{Input}}$$

(4) $$\text{WCR}\;\%=\frac{\left(\text{PWC}\;-\;\text{PPWC}\right)*100}{\;\text{PWC}}$$

where: WCR = Percentage of work content reduction per piece.

PWC = Present work content per piece.

PPWC = Proposed content per piece.

The stacking of raw materials far from machine M1 and the lack of a table to stand at a comfortable height to perform the punching operation were found to be the main drawbacks of the layout of the existing machines and the process. Additionally, there is no adequate space at the end of machine M3 to properly stack the products that have been produced. To reduce errors, a batch of 10 pieces underwent a time study and the average time is taken as the time required per piece. After measuring the location of the raw material, it was found that ×1 = 1.23 m and Y1 = 1.65 m. Additionally, the intermediate product was stacked at positions ×2 = 1.05 m and Y2 = 1.55 m (Fig. 7). For comparison purposes, only the middle batch raw material and the intermediate product are considered when determining the operating height.

The new machine layout is planned for the placement of both the raw materials and the intermediate products, taking into account the process, the location of jigs, and dimensional parameters, ensuring that the operators (O1, O2, O5 and O6) move less and subjected to less fatigued as a result. The values of ×1 = ×2 = Y1 = Y2 = 1 m (Fig. 7) for the raw material as well as in intermediate product side to provide sufficient space for the operators fee movement. By leaving one batch of the raw material as dead stock on both sides of the raw material and the intermediate product, the operating height range is set between 0.625 m and 1.05 m. The lifting and putting of both raw materials and intermediate products at a lower height by operators (O1, O2, O5 and O6) greatly increases production capacity by reducing extra movement.

### 4.2 Production increase

Without implementing scientific method, the company's production supervisors set a daily production capacity target of 420 pieces by referring to the previous time daily production report sheet. Moreover, they stated during the interview, to verify the achievability of this target they assigned skilled operators who had the fabrication experience of using the machineries in previous similar orders. But, according to the daily report sheet, they could not able to achieve the target of 420 pieces per day (8 h) in most of the time and furthermore their performance was inconsistent. If a time study were done to set the production target, the target daily production capacity of the electric pole cross arm fabrication would be 443 pieces (Table 5), which is still higher than what they set and lower than what they actually produced. The new layout allows them for proper placement of raw materials and intermediate products have contributed to increment of productivity by reducing idle travel during feed of the raw materials and stacking of the intermediate products. Furthermore, under the previous arrangement, the workers had to feed the raw materials from the height of 0.1 m–1.575 m off the ground, making it difficult to handle a 22 kg work piece within this range of height for the operators (O1, O2, O5 and O6).

**Table 5:** Existing product cycle time (baseline cycle time).

Testing Time	Trial	Measured time for 10 consecutive pieces [Time in second]	Calculated time taken per piece [Time in second]	Standard Deviation	Calculated 8hr. production capacity
9:00AM-12:00AM	1	641	64		
	2	663	66		
	3	620	62	**1.793**	
	4	620	62		
	5	641	64		
**Average time taken (mean)**		**637**	**64**		**450**
2:00PM- 5:00PM					
	1	630	63		
	2	672	67		
	3	663	66	**1.517**	
	4	649	65		
	5	662	66		
**Average time taken/Production**		**665**	**66**		436
**Grand average**		**646**	**65**		443

Operators will become extremely exhausted as a result, which will lower performance. The two stacks, each of which contains 100 pieces, are left as a dead stack and used as an operating table to change the operating height range. With the new setup the operating height range is changed to 0.625 m–1.05 m. Consequently, setting to ergonomically suitable working height greatly increases production capacity. With the three punching machines that are currently in use, with 6 people (two people per machine), the company's production capacity has increased from daily target output of 420 pieces to 720 pieces (actual measured value) in 8 h. The work study predicted that the average capacity would be 738 pieces per day (Table 6). However, in practice, the operators fabricated 720 pieces per day, which is 97.6 % of the predicted value from the work study. Consequently we can conclude that the work study method correctly estimated the operators' actual production efficiency in the new configuration.

**Table 6:** Improved product cycle time.

Testing Time	Batch	Measured time taken for 10 consecutive piece [Time in second]	Calculated time taken per piece [Time in second]	Standard Deviation	Calculated 8hr. production capacity
9:00AM-12:00AM	1	399	40		
	2	338	34		
	3	379	38	**3.536**	
	4	370	37		
	5	306	31		
**Average time taken/Production**		**358**	**36**		**800**
2:00PM- 5:00PM					
	1	441	44		
	2	450	45		
	3	454	45	**3.209**	
	4	375	38		
	5	400	40		
**Average time taken/Production**		**424**	**42**		**685**
**Grand average**		**391**	**39**		**735**

### 4.3 Reduce idle time

By removing unnecessary U-channel travel for loading and unloading, the company's productivity has increased by lowering the machine's idle time. This contributes to shorten delivery date of the product to the customer as a result it will raise to customer satisfaction. The total cycle time to produce one product is lowered by 26 s, saving 11,518 s (3 h and 12 min) in one day (8 h). This reduction in time required for the fabrication greatly improves the company overall performance and profit margin.

### 4.4 Reduce worker fatigue

Focus group discussion was held before and after the improvement of the layout with a group of six workers who are in charge of the production process. The four operators (O1, O2, O5 and O6) qualitatively proven that they are at ease with the new arrangement. Furthermore, they are encouraged to manufacture more than the assigned quantity by the company with the given time. They realize that the previous arrangement made it challenging for them to operate the machines and exposed them to morning back pain. In particular those who operate the first and third machines explained how they are now much more comfortable as a result of the reduction of the idle travel and convenient height range that would have otherwise caused back pain during raw material loading and intermediate product stacking.

## 5 Conclusion and recommendation

It has been noted that the use of work study techniques has improved layout and cut cycle time, eliminating unneeded motions of the material and lowering worker fatigue. Without any additional capital investment, such as purchasing a new machine, it has increased the productivity of the workers by 71 % and this will improves the profit of the company by producing of greater number products which is raised from 420 pieces to 720 pieces per day. Due to shorter delivery times, this improvement will boost the company's overall performance and leads to customer satisfaction. The result obtained from this research indicated that the implementation of new layout for the raw material and for the intermediate product reduce the cycle time of 26 s which leads to rise in productivity by additional 300 pieces more. The new arrangement resulted in a significant reduction in the workload for the operators (O1, O2, O5, and O6) working on M1 and M3, while the workload for the two operators (O3 and O4) working on M2 remained constant. The work study has much further scope and potential for enhancing productivity by making changes in the jigs design, machine layout and enhancing ergonomics of work environments. Moreover, designing a new layout is needed by considering the fourth punching machine which now becoming the bottleneck for the overall productivity improvement. During the study, the punching machine repeatedly broke down, so the maintenance practice must be studied, which also contributes to productivity improvement.

The findings can also be used to improve the scheduling of the fabrication process and to develop incentive schemes in which employees receive bonuses and wage increases if they meet their goals and deadlines. This could enhance overall productivity and create a more dedicated workplace.

## Data availability statement

Data included in this article.

## Funding

Bahir Dar Technology Institute at Bahir Dar University in Ethiopia financed this research (staff externship program), which covered one month's lodging and travel expenses.

## CRediT authorship contribution statement

**Yonas Mitiku Degu:** Writing – review & editing, Writing – original draft, Validation, Methodology, Investigation, Funding acquisition, Formal analysis, Data curation, Conceptualization.

## Declaration of competing interest

The authors declare the following financial interests/personal relationships which may be considered as potential competing interests:Yonas Mitiku Degu reports financial support and travel were provided by Bahir Dar Institute of Technology, 10.13039/501100005872Bahir Dar University. Yonas Mitiku Degu reports a relationship with Bahir Dar Institute of Technology, Bahir Dar University that includes: employment. If there are other authors, they declare that they have no known competing financial interests or personal relationships that could have appeared to influence the work reported in this paper.
